# Memory T Cells Generated by Prior Exposure to Influenza Cross React with the Novel H7N9 Influenza Virus and Confer Protective Heterosubtypic Immunity

**DOI:** 10.1371/journal.pone.0115725

**Published:** 2015-02-11

**Authors:** Sean R. McMaster, Jon D. Gabbard, Dimitris G. Koutsonanos, Richard W. Compans, Ralph A. Tripp, S. Mark Tompkins, Jacob E. Kohlmeier

**Affiliations:** 1 Department of Microbiology and Immunology, Emory University School of Medicine, Atlanta, Georgia, United States of America; 2 Department of Infectious Diseases, College of Veterinary Medicine, University of Georgia, Athens, Georgia, United States of America; The University of Chicago, UNITED STATES

## Abstract

Influenza virus is a source of significant health and economic burden from yearly epidemics and sporadic pandemics. Given the potential for the emerging H7N9 influenza virus to cause severe respiratory infections and the lack of exposure to H7 and N9 influenza viruses in the human population, we aimed to quantify the H7N9 cross-reactive memory T cell reservoir in humans and mice previously exposed to common circulating influenza viruses. We identified significant cross-reactive T cell populations in humans and mice; we also found that cross-reactive memory T cells afforded heterosubtypic protection by reducing morbidity and mortality upon lethal H7N9 challenge. In context with our observation that PR8-primed mice have limited humoral cross-reactivity with H7N9, our data suggest protection from H7N9 challenge is indeed mediated by cross-reactive T cell populations established upon previous priming with another influenza virus. Thus, pre-existing cross-reactive memory T cells may limit disease severity in the event of an H7N9 influenza virus pandemic.

## Introduction

Influenza viruses are a primary cause of severe respiratory tract infections worldwide. In addition to the health risks from circulating seasonal strains of influenza, the potential for antigenic shift and emergence of zoonotic pandemic strains present significant risks for increased morbidity and mortality. In February of 2013, an H7N9 influenza A virus (H7N9) of avian origin was laboratory confirmed in four human cases, resulting in three deaths [1]. Clinical symptoms in these cases included fever and intractable pneumonia unresponsive to antibiotics, which progressively extended to more severe systemic complications [2]. The majority of human cases have been associated with either direct contact with avian sources or poultry markets, and transmission studies on H7N9 in guinea pigs and ferrets have demonstrated a limited ability to effectively transmit via respiratory droplets suggesting that H7N9 has not achieved sustained human-to-human transmission [3–6]. However, recombination with other influenza viruses with greater propensity to bind human respiratory epithelium would be a cause for concern [7], and a recent surveillance study showed evidence for increased pandemic potential of H7N9 following an outbreak of 127 confirmed cases during January 2014, ten cases fewer than all of the 2013 season [1,8].

Antibodies generated against circulating influenza viruses following infection or vaccination do not convey neutralizing protection against the novel H7N9 virus [9]. In contrast, two initial studies showed that PBMCs from healthy donors expanded *in vitro* contained T cells that were cross-reactive for H7N9-derived target peptides or infected cells [10,11]. This is important, as animal models have shown that influenza-specific memory T cells can confer protection against a lethal challenge from an unrelated influenza virus in the absence of neutralizing antibody [12]. Furthermore, a recent study demonstrated an important role for CD8 T cell (CTL) heterosubtypic immunity in decreasing clinical complications in humans during the 2009 H1N1 influenza pandemic [13]. Thus, it is conceivable that cross-reactive influenza-specific memory T cell responses could similarly convey a milder clinical course and reduced morbidity during H7N9 infection.

The potential of pre-existing cellular immunity to influenza virus to provide some degree of protection against H7N9 influenza infection is currently unknown because we do not know the level of memory T cell cross-reactivity between common circulating strains and H7N9, nor have animal models addressed the potential for heterosubtypic immunity to protect against a lethal H7N9 challenge. In the current study, we investigated the native frequency of H7N9 cross-reactive memory T cells in mice following infection with common laboratory influenza strains and in human PBMCs from healthy donors. Our data show that there is a significant percentage of memory T cells that recognize H7N9 influenza in mice following infection with several different common influenza strains and also in human PBMCs from healthy donors. We have expanded previous studies investigating human CD8 T cell cross-reactivity to H7N9 influenza virus [10,11] by demonstrating that there is also significant human CD4 T cell cross-reactivity and by demonstrating that cross-reactivity to H7N9 in humans is sufficiently robust to be identified by using *ex vivo* analyses instead of skewing populations through *in vitro* expansion of target populations. Furthermore, cross-reactive memory T cells in mice were able to confer protection from a lethal H7N9 challenge and led to more rapid viral clearance. Thus, our data suggest that cross-reactive memory T cell responses may play an important role in limiting the severity of H7N9 infection in humans.

## Materials and Methods

### Mice

C57BL/6J mice from The Jackson Laboratory were housed under specific ABSL2 conditions at Emory University. For H7N9 challenge studies at the University of Georgia, mice were housed under ABSL2 conditions for immunization and then transferred to ABSL3 facilities for H7N9 challenge.

### Ethics Statement

All experiments in this study were approved and completed in accordance with the Institutional Animal Care and Use Committee guidelines of Emory University (Protocol Number: DAR-2001547–071315GN) and the University of Georgia (Protocol Number: A2014 04-025-Y1-A0). The above named Institutional Animal Care and Use Committees specifically approved this study. All efforts were made to minimize suffering.

### Influenza infections

Intranasal infections with influenza A/HKx31 (H3N2), A/PR8 (H1N1) and pandemic A/California/09 (H1N1) were used to generate influenza virus-specific memory T cells in mice as previously described [14]. Influenza A/PR8 (H1N1) was used to establish immunological memory prior to secondary challenge with either 0.5 mLD_50_ (mouse LD_50_) or 5 mLD_50_ influenza A/Anhui/1/2013 (H7N9). After H7N9 challenge, mice were monitored for weight loss and clinical symptoms every other day. Animals reaching defined endpoints of less than 65% original weight were humanely euthanized by Tribromoethanol (Avertin) overdose (600mg/kg) followed by brachial exsanguination. No other analgesics or anesthetics were administered during the time course. Subsets of mice were humanely euthanized on days three and six post-H7N9 challenge for analysis of lung virus titers. A/Anhui/1/2013 (H7N9) was provided by Richard Webby (St. Jude Children’s Research Hospital, Memphis, TN) through the WHO Global Influenza Surveillance and Response System (GISRS) and propagated in embryonated chicken eggs as previously described [15]. All work with H7N9 influenza was conducted in BSL3 or ABSL3 facilities following protocols approved by the University of Georgia Institutional Biosafety Committee.

### Viral antibody cross-reactivity

Bronchoalveolar lavage supernatants and serum were individually collected from PR8 memory mice, and anti-H7N9, anti-PR8, anti-X31 specific IgG antibody levels were determined quantitatively by enzyme-linked immunosorbent assay (ELISA) as previously described [16], using whole inactivated virus. Purified mouse IgG and goat anti-mouse-HRP for ELISA were purchased from Southern Biotechnology Associates. Optical density was read at 450nm.

### Human PBMCs

Eleven de-identified human PBMCs purchased from Cellular Technology Limited were chosen on the basis of positive reactivity (as measured by IFN-γ ELISpot performed by Cellular Technology Limited) to peptides of common influenza virus T cell epitopes, described in Table 1. Patient demographic data delineated in Table 2. The country of origin of the human PBMC samples was the United States of America with ten of the eleven samples being collected between May 2006 and September 2012; a single patient in our study donated PBMCs early in February 2013.

**Table 1:** MHC Class I peptides from CTL to screen for previous exposure to influenza virus of human PBMC samples denoted in Fig. 4.

CTL Peptide Number	Virus, Protein_region_	HLA-Allele	Peptide sequence
**CEF-1**	Influenza A, PB-1_591–599_	HLA-A1	VSDGGPNLY
**CEF-2**	Influenza A, NP_44–52_	HLA-A1	CTELKLSDY
**CEF-3**	Influenza A, M1_58–66_	HLA-A2	GILGFVFTL
**CEF-4**	Influenza A, PA_29–37_	HLA-A2	FMYSDFHFI
**CEF-8**	Influenza A, NP_91–99_	HLA-A68	KTGGPIYKR
**CEF-9**	Influenza A, NP_342–351_	HLA-A3	RVLSFIKGTK
**CEF-10**	Influenza A, NP_265–274_	HLA-A3	ILRGSVAHK
**CEF-13**	Influenza A, M1_13–21_	HLA-A3/A11/A6	SIIPSGPLK
**CEF-18**	Influenza A, NP_418–426_	HLA-B7	LPFDKTTVM
**CEF-20**	Influenza A, NP_380–388_	HLA-B8	ELRSRYWAI
**CEF-25**	Influenza A, NP_383–391_	HLA-B27	SRYWAIRTR
**CEF-26**	Influenza A, M1_4–11_	HLA-B27	ASCMGLIY

**Table 2:** Human patient demographic data for samples denoted in Fig. 4.

Patient	Age	Gender	Ethnicity
**1**	36	Male	Caucasian
**2**	39	Male	Caucasian
**3**	49	Male	African American
**4**	36	Male	Caucasian
**5**	40	Male	Caucasian
**6**	27	Male	Caucasian
**7**	36	Female	Hispanic
**8**	35	Male	Hispanic
**9**	25	Female	Hispanic
**10**	33	Male	Hispanic
**11**	26	Male	Filipino

### Cellular stimulation & intracellular cytokine staining (ICS)

Whole virus was heat-inactivated {Sendai virus, influenza A/HKx31 (H3N2), A/PR8 (H1N1) and pandemic H1N1} or β-propiolactone inactivated {H7N9} [15] and used separately to stimulate human PBMCs or mouse lung-derived lymphocytes for 18 hours. Brefeldin A was added during the last four hours of murine cell stimulation; Monensin was added with Brefeldin A to the human PBMCs for this time period. Following stimulation, human PBMCs were then stained with Zombie NIR (BioLegend) to exclude dead cells. Staining for intracellular cytokines was performed as previously described [14].

### Flow cytometry

Monoclonal antibodies used to stain human PBMCs were from BioLegend CD27 (O323), CD8α (RPA-T8), CD3 (OKT3), IFN-γ (4S.B3), TNFα (MAb11), and BD Biosciences CD4 (RPA-T4). Monoclonal antibodies used to stain lung-derived murine lymphocytes were BioLegend CD3 (17A2), CD8α (53–6.7), CD4 (RM4–5); eBioscience CD11b (M1/70), CD44 (IM7); and BD Biosciences IFN-γ (XMG1.2). Samples were run on a BD Biosciences LSRII flow cytometer and analyzed with FlowJo software.

### Viral titers

Tissue titers were determined as previously described [15,17]. Briefly, lungs were homogenized in 1mL PBS and cleared by centrifugation. Supernatants were titrated on MDCK cells in MEM containing 1μg/mL TPCK-trypsin (Worthington) and cultured for 72 hours. Supernatants were assayed for presence of influenza virus by hemagglutination using 0.5% chicken RBCs.

### Statistical analysis

All analysis was performed in GraphPad Prism 6. One-tailed paired t tests were used to test significance of human and mouse ICS data when comparing H7N9 stimulated conditions to either the paired unstimulated or Sendai virus stimulated control. One-way ANOVA was used to analyze ELISA data with corrected multiple comparisons being evaluated for significance by Tukey’s test. Two-tailed t tests were used to evaluate significance of viral titer data and mouse weight loss after Holm-Sidak correction for multiple comparisons. Differences in survival over the 14 day challenge period were evaluated for significance using the Logrank Mantel-Cox test.

## Results

### Cross-reactive CD4 and CD8 responses to H7N9 influenza virus in mice

**Fig 1:**
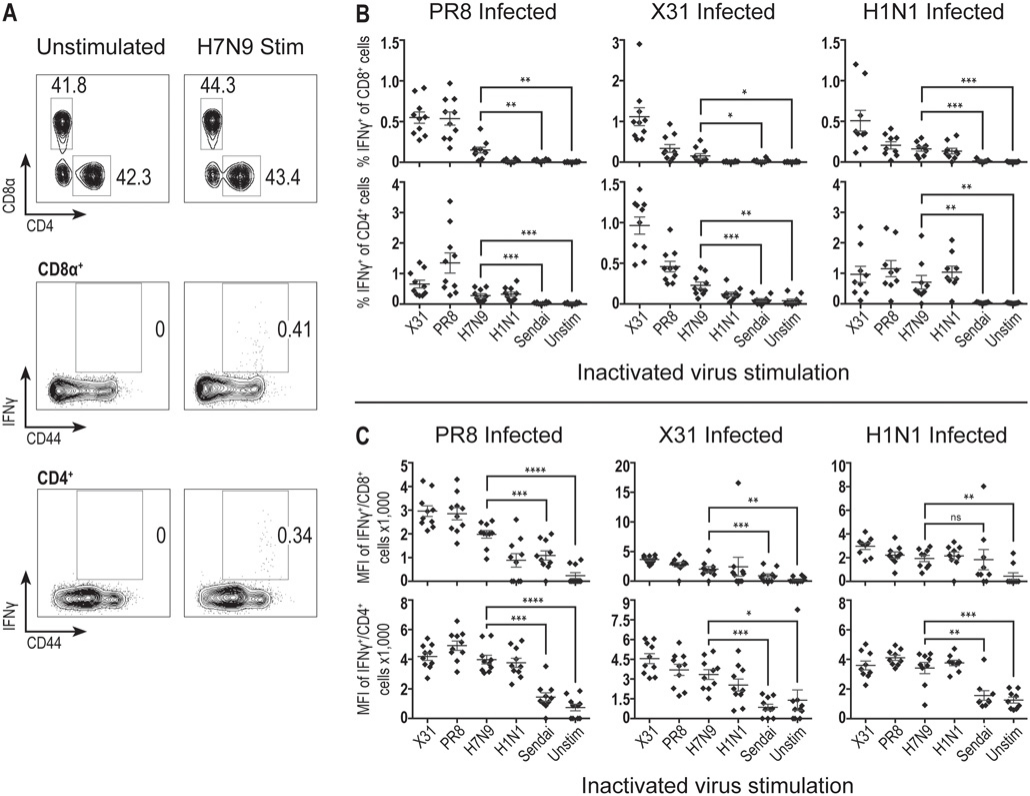
H7N9 is recognized by memory CD4 and CD8 T cells derived from prior influenza virus exposure in mice. (A) Representative IFN-γ expression frequency in mouse CD8^+^ and CD4^+^ cells either unstimulated or stimulated for 18 hours with whole inactivated H7N9 influenza virus. CD8^+^ and CD4^+^ cells gated on a CD11b^-^/CD3ε^+^ population and derived from mouse lungs infected with 600 EID_50_ PR8 and rested 35 days. (B) IFN-γ frequency in mouse CD8^+^ {top} and CD4^+^ {bottom} cells following 18 hours of stimulation with either whole inactivated influenza virus {X31, PR8, H7N9, pH1N1}, whole inactivated Sendai virus, or left unstimulated. Cells for intracellular cytokine staining (ICS) were derived from mouse lungs 35 days post-infection with 600 EID_50_ PR8, 30,000 EID_50_ X31, or 1326 EID_50_ pH1N1 influenza virus. N = 19–20 mice pooled from two experimental replicates; mean and standard error of the mean displayed. Representative of seven experiments at memory and acute time points. (C) Mean fluorescence intensity of IFN-γ^+^/CD8^+^ {top} or IFN-γ^+^/CD4^+^ {bottom} cells whose frequencies were displayed in B. Statistics: One-tailed paired t tests used in 1B & C to compare H7N9 stimulated samples to either unstimulated or Sendai stimulated matched samples; p-values {* <0.05; ** <0.01; *** <0.001; **** <0.0001}.

We sought to quantify the frequency of H7N9 cross-reactive T cells under controlled influenza virus exposure. Thus, we infected mice with influenza A/HKx31 (X31), A/PR8 (PR8), or pandemic A/California/09 H1N1 (pH1N1) to evaluate the individual predisposition of each infection to generate H7N9 cross-reactive T cells. We harvested lymphocytes from the lungs of mice 35 days post-infection and stimulated cells with whole inactivated virus (X31, PR8, H7N9, pH1N1, or Sendai virus) to evaluate T cell reactivity via intracellular cytokine staining. CD8 and CD4 T cells were examined independently for the production of IFN-γ in response to virus stimulation (Fig. 1A). IFN-γ responses to inactivated H7N9 influenza virus stimulations were directly compared to paired samples that were either left unstimulated or were stimulated with inactivated Sendai virus as a negative control, as Sendai virus does not share any cross-reactive T cell epitopes with influenza virus. For mice infected with each of the three priming conditions (PR8, X31, pH1N1), there was a significant percentage of CD8 (Fig. 1B, top) and CD4 (Fig. 1B, bottom) T cells cross-reactive to H7N9 as compared to matched unstimulated or Sendai virus stimulated samples. As expected, for all three priming conditions, the CD4 and CD8 T cell cross-reactivity to H7N9 was less than that observed following stimulation with homologous inactivated virus or heterologous inactivated virus with identical internal proteins (PR8 and X31). Moreover, for all three priming conditions, examination of the geometric Mean Fluorescence Intensity (MFI) for IFN-γ following H7N9 stimulation was found to be significant compared to the matched unstimulated samples of both CD8 (Fig. 1C, top) and CD4 (Fig. 1C, bottom) T cells. When comparing IFN-γ MFI of H7N9 stimulated to Sendai virus stimulated samples, only the CD8 T cell population of PR8- and X31-primed mice stimulated with H7N9 were found to be significantly higher than the Sendai virus stimulation (Fig. 1C, top); looking at CD4 IFN-γ MFI, all three priming conditions were found to produce a CD4 T cell population where the H7N9 stimulated samples have a significantly greater IFN-γ MFI as compared to those stimulated with Sendai virus (Fig. 1C, bottom).

### Limited anti-H7N9 cross-reactive antibody in PR8 memory mice

**Fig 2:**
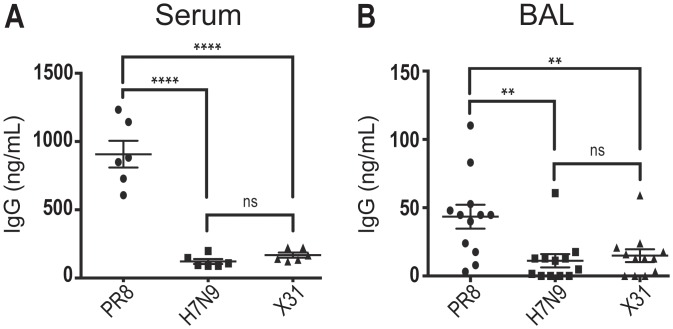
Limited antibody cross-reactivity between PR8 and H7N9 influenza viruses. (A) Concentration of anti-PR8, anti-H7N9, and anti-X31 specific IgG antibody levels in the serum of mice previously infected with PR8 and rested to immunological memory. N = 6; mean and standard error of the mean displayed. (B) Concentration of anti-PR8, anti-H7N9, and anti-X31 specific IgG antibody levels in the supernatant of a bronchoalveolar lavage (BAL) from mice previously infected with PR8 and rested to immunological memory. N = 12; mean and standard error of the mean displayed. Statistics: One-way ANOVA was used to compare concentrations of cross-reactive IgG, multiple comparisons, with correction, were evaluated for significance by Tukey’s test; p-values {** <0.01; **** <0.0001}.

To confirm that priming with PR8, an H1N1 virus, did not generate any antibodies that recognized H7N9, we performed an ELISA on serum from PR8 memory mice. As expected in PR8-primed mice, we detected a significantly lower concentration of H7N9-reactive IgG antibody in the serum as compared to the concentration of PR8-reactive IgG antibody; in fact, the level of H7N9-reactive IgG was statistically similar to that of X31-reactive IgG (Fig. 2A), which is known to be serologically distinct from PR8 [18]. Furthermore, this observation held when we looked at the cross-reactive antibody concentrations in the lung airways, as determined by bronchoalveolar lavage (BAL), of PR8-primed mice. Anti-H7N9 cross-reactive IgG antibody levels in PR8-primed lung airways were statistically similar to levels of anti-X31 cross-reactive IgG levels, both of which were significantly lower than the levels of PR8-reactive IgG antibody (Fig. 2B).

### Memory T cells from previous influenza virus exposure protect against H7N9 challenge

**Fig 3:**
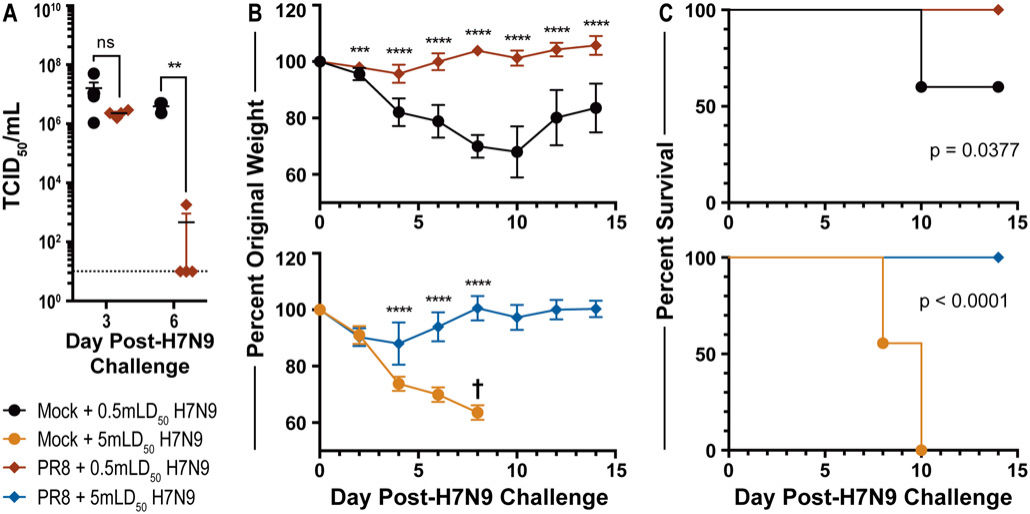
Memory T cell responses generated following PR8 infection protect against lethal H7N9 challenge. (A) H7N9 influenza viral titers on day three or day six following challenge with 0.5 mouse LD_50_ (mLD_50_) H7N9. Mice were previously mock-primed or PR8-primed (600 EID_50_) and rested for 42 days prior to H7N9 challenge. N = 4–5 mice per day per group; mean and standard error of the mean displayed; representative of one experiment. Dotted line denotes ten as the level of detection. (B) Weight loss over 14 days of mice challenged with 0.5 mLD_50_ (top) or 5 mLD_50_ (bottom) H7N9. Mice were primed and rested per A. † denotes all mice from 5 mLD_50_ H7N9 challenge group were dead or euthanized by day eight post-H7N9 challenge. N = 9–10 mice per group; mean and standard deviation displayed; representative of one experiment. (C) Survival curve of mice challenged with 0.5 mLD_50_ (top) or 5 mLD_50_ (bottom) H7N9 over 14 days; same mice for which weight loss was measured in B. Mice were primed and rested per A. N = 9–10 mice per group; representative of one experiment. Statistics: Two-tailed t tests used in 3A-B to compare mock-primed and PR8-primed groups following H7N9 challenge; 3B t tests corrected for multiple comparisons using Holm-Sidak correction; survival differences in 3C evaluated with the Logrank Mantel-Cox test; p-values {** <0.01; *** <0.001; **** <0.0001}.

Given that the native frequency of H7N9 cross-reactive T cells was substantial in mice previously exposed to influenza virus, we wanted to know whether these memory T cells were able to convey protection upon challenge with H7N9. We tested this question by using mice mock-infected or primed with PR8 and rested to immunological memory before challenging with H7N9. We evaluated the infectious burden of the H7N9 0.5 mouse LD_50_ (mLD_50_) challenge in PR8-primed and mock-primed mice by measuring viral titers at days three and six post-secondary challenge. While viral titers were not significantly different at day three post-H7N9 challenge between the mock-primed and PR8-primed mice, we did find a significant difference in titers at day six post-challenge (Fig. 3A). This could infer that the cross-reactive memory T cells in the PR8-primed mice enabled a much more rapid clearance of H7N9 virus, despite the original infectious burden at day three remaining relatively the same between the two groups.

We also assessed clinical manifestations of the disease course by measuring weight loss and survival for 14 days following infection with 5 mLD_50_ or 0.5 mLD_50_ H7N9. We found large and significant divergences between the PR8-primed and the mock-primed groups four days following H7N9 challenge (Fig. 3B); this divergence continued to grow until about day ten post-H7N9 challenge for the 0.5 mLD_50_ dose or until all mock-primed mice of the 5 mLD_50_ dose were dead by day eight post-H7N9 challenge (†). All PR8-primed mice survived challenge with either 0.5 mLD_50_ or 5 mLD_50_ H7N9, whereas all mock-primed mice from the 5 mLD_50_ group and 40% from the 0.5 LD_50_ group died or were euthanized by day ten (Fig. 3C). For the 0.5 mLD_50_ and 5 mLD_50_ challenge, the results between the PR8-primed and mock-primed groups were found to be statistically significant with p-values of 0.0377 and <0.0001, respectively.

### CD4 and CD8 T cells derived from humans with previous exposure to influenza virus exhibit cross-reactivity to H7N9

**Fig 4:**
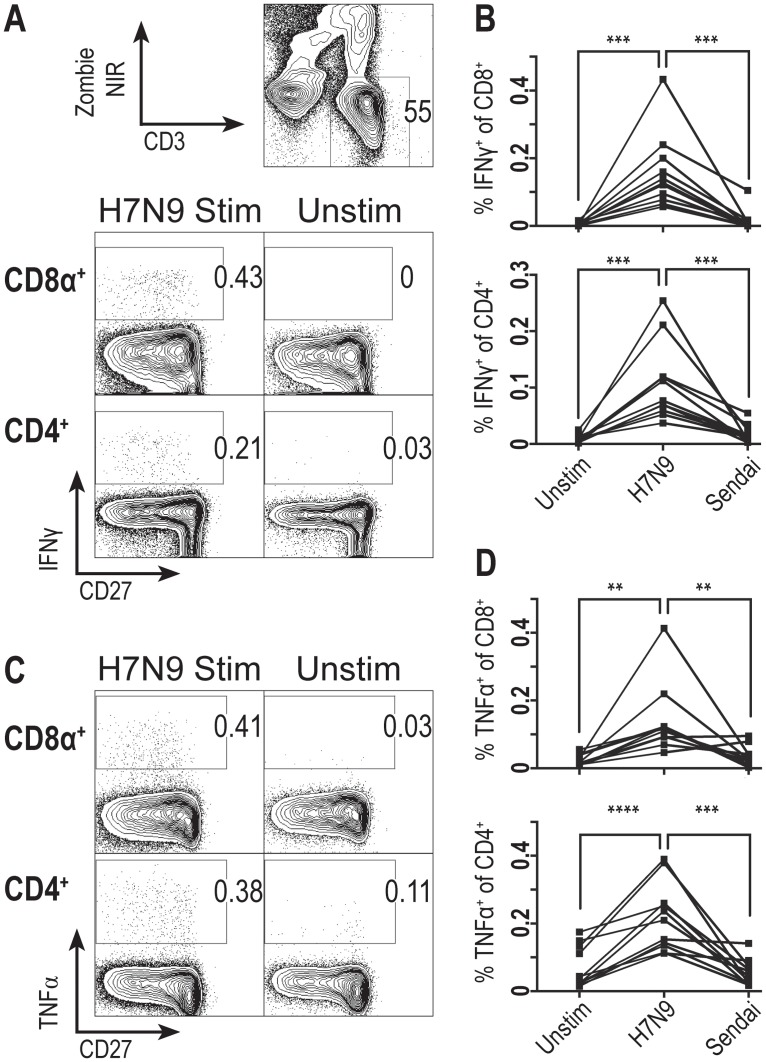
H7N9 is recognized by memory CD4 and CD8 T cells derived from humans with prior influenza virus exposure. (A) Representative IFN-γ expression frequency in human CD8^+^ and CD4^+^ cells either unstimulated or stimulated for 18 hours with whole inactivated H7N9 influenza virus. CD8^+^ and CD4^+^ cells gated on a ZombieNIR^-^/CD3^+^ population and derived from human PBMC samples purchased from Cellular Technology Limited. (B) IFN-γ frequency in live human CD8^+^ {top} and CD4^+^ {bottom} cells following 18 hours of stimulation with either whole inactivated H7N9 influenza virus, whole inactivated Sendai virus, or left unstimulated. N = 11; stimulations for individual patients are interconnected with lines. (C) Representative TNF-α expression frequency in human CD8^+^ and CD4^+^ cells either unstimulated or stimulated for 18 hours with whole inactivated H7N9 influenza virus, gated as described in A. (D) TNF-α frequency in live human CD8^+^ {top} and CD4^+^ {bottom} cells described in C. Statistics: One-tailed paired t tests used in 4B & D to compare H7N9 stimulated samples to either unstimulated or Sendai stimulated samples; p-values {** <0.01; *** <0.001; **** <0.0001}.

Our findings on the level of H7N9 cross-reactivity in mice suggested that we might be able to detect the native frequency of H7N9 cross-reactive T cells in human samples. Thus, we procured human PBMCs from healthy donors with previous exposure to influenza virus; demographic data is provided in Table 2. Following stimulation with inactivated H7N9 virus, there was a significant increase in IFN-γ production compared to paired samples left unstimulated or stimulated with inactivated Sendai virus in both CD8 and CD4 T cell populations, when gating on live, CD3^+^ cells (Fig. 4A & 4B). Moreover, this significance also held when looking at TNF-α production in both CD8 and CD4 T cell populations when comparing paired samples stimulated with inactivated H7N9 virus to either unstimulated or inactivated Sendai virus stimulated samples (Fig. 4C & 4D). The presence of cross-reactive T cells to H7N9 in humans could portend similar protection to what we observed with our murine H7N9 challenge.

## Discussion

While nearly all of the identified cases of human H7N9 infection have occurred as a result of direct or indirect interaction with avian sources, there is a risk that the virus could gain the capacity to effectively transmit between humans. Given the absence of widespread human exposure to either H7 or N9 influenza viruses, any type of pre-existing immunological protection would likely be derived from cross-reactive cellular immunity against other influenza virus strains. Because of this, our findings have broad implications in regard to previous influenza A virus exposure and development of cross-reactive T cells as a protective correlate to the emerging H7N9 influenza virus. We demonstrate that there exists a cross-reactive CD4 and CD8 memory T cell population found in both humans and mice able to recognize and produce antiviral cytokines in response to H7N9 exposure. Moreover, our H7N9 challenge study provides direct evidence that the existence of cross-reactive memory T cells from previous exposure to influenza A viruses, in our case PR8, correlates with reduced morbidity and mortality in the murine model. Furthermore, our results of limited antibody cross-reactivity between PR8 and H7N9 suggest that neutralizing antibodies are most likely not the source of the observed protection, affording additional credence that the protection from H7N9 challenge is mediated by pre-existing H7N9 cross-reactive T cells established from previous influenza virus exposure.

One could posit that such a correlation would also hold true for humans, as cross-reactive memory T cells could convey protection to H7N9 infection by limiting viral replication during the early stages of the immune response and thus limit the clinical manifestations of the infection. This is important since cross-reactive neutralizing antibodies generated by exposure to other circulating influenza viruses and capable of effectively targeting H7N9 have not been identified in the general human population, nor in poultry workers in China [19,20]; furthermore, H7 influenza A viruses have been characterized as being poorly immunogenic, including the emerging H7N9 virus where poor antibody generation and helper T cell function is predicted in humans [21]. Therefore, in juxtaposition with the finding that CTL responses aided in reducing morbidity during the 2009 H1N1 influenza pandemic [13], perhaps CTL responses could once again aid in limiting both morbidity and mortality in the event of an H7N9 influenza virus pandemic.

## Supporting Information

S1 ARRIVE Checklist ARRIVE checklist of information included in this article. (PDF)
